# Criteria for Routine Laboratory Blood Tests in Patients Hospitalized in Cardiology Departments

**DOI:** 10.3390/diagnostics15182307

**Published:** 2025-09-11

**Authors:** Zvi Shimoni, Fadi Hin, Paul Froom

**Affiliations:** 1 The Adelson School of Medicine, Ariel University, Ariel 4070000, Israel; zshimoni@laniado.org.il; 2 Sanz Medical Center, Laniado Hospital, Netanya 4244916, Israel; 3 Cardiology Department, Sanz Medical Center, Laniado Hospital, Netanya 4244916, Israel; toti_fhin@hotmail.com; 4 Clinical Utility Department, Sanz Medical Center, Laniado Hospital, Netanya 4244916, Israel; 5 School of Public Health, University of Tel Aviv, Tel Aviv 6997801, Israel

**Keywords:** criteria, routine, laboratory tests, cardiology department

## Abstract

The proportion of laboratory tests ordered in cardiology departments without clinical utility is unclear. The objective of this study was to determine if criteria limiting testing can safely reduce admission and follow-up testing. We reviewed the charts of 471 consecutive patients admitted to the cardiology department at a regional hospital from January 2019 to June 2019. We prospectively set appropriate criteria for routine admission and follow-up testing. Commonly ordered tests and parameters considered not to be indicated either on admission or on follow-up included C-reactive protein, liver function tests, lactate dehydrogenase, creatine phosphokinase, calcium, blood urea nitrogen, uric acid, cholesterol, Hemoglobin A1c, and prothrombin times (except for patients treated with warfarin). Admission tests considered appropriate included electrolytes, glucose, creatinine, and complete blood counts. Follow-up testing was indicated only if test results were outside the reference ranges. Troponin tests were only indicated if needed to determine the need for a coronary angiogram. The outcome variables were the proportion of indicated tests and whether tests outside the criteria led to changes in acute care that positively affected the patient’s hospital care. In the 471 patients, there were 18,061 tests ordered (not including troponin), and 14,427 (79.9%) were not indicated; this led to 46 (0.3%) changes in medical care, which did not affect the patients’ clinical course. There were 47.8% (364/761) troponin tests that were not indicated and did not change patient care. Our study suggests that interventions in cardiology departments such as ours could safely reduce troponin testing by nearly 50% and other laboratory tests by around 80%. These results need to be confirmed in other settings and in interventional studies.

## 1. Introduction

Excessive laboratory blood testing increases direct costs and the personnel required to draw the blood and identify, execute, and validate the test results. Furthermore, excessive testing may even prolong hospital stays and result in clinical disutility if the results are misinterpreted [1,2,3,4].

The definitions of laboratory over-testing, however, are varied, and to effectively address unnecessary testing, it is crucial to define what constitutes inappropriate utilization [5,6] and demonstrate that reducing laboratory testing is safe. A recent meta-analysis [7] included studies that assessed interventions including computerized provider order entry, clinical decision support systems/tools, education, and auditing with feedback. The interventions were variably effective and did not increase the rates of patient-important outcomes assessed (e.g., morbidity, mortality, and length of stay). However, directly demonstrating that omitted tests would not have changed medical management is a more sensitive way to ensure that reduced testing is safe.

There are only a few studies reporting changes in medical care after reductions in testing; one included as acceptable all tests relevant to the patient’s symptoms, or potentially dictating the ordering of new laboratory tests [8], and another included laboratory and imaging tests [9]. Recently, a study of patients with community-acquired pneumonia reported that excluding tests that did not change medical care would decrease testing by over 80% [10]. We are unaware of previous studies done in cardiology departments.

In this historical prospective study, we reviewed the medical records of consecutive patients hospitalized in a cardiology department. After establishing criteria for admission and follow-up tests, we determined if restricting testing would miss appropriate changes in acute medical care.

## 2. Materials and Methods

We reviewed the charts of 471 consecutive patients admitted directly from the emergency department to the cardiology department at a regional hospital from January 2019 to June 2019, and included patients with acute disease or referred for an elective procedure. The number of commonly ordered basic admission and follow-up laboratory tests was extracted from the electronic database. We prospectively set criteria for admission testing based on prior recommendations [10] (Table 1). There are no national standards for ordering laboratory tests. Follow-up tests were indicated only if admission testing results were outside the reference ranges, except for complete blood counts that had specific criteria (Table 1). Troponin tests were considered indicated in patients with a suspected myocardial infarction except in those with a ST-elevated myocardial infarction (STEMI) or new onset congestive heart failure because treatment is the same regardless of the test results. A high-sensitivity cardiac troponin T assay (Roche CARDIAC Trop T Sensitive; Roche, Basel, Switzerland) was used.

Prothrombin (PT-INR) testing before anticoagulation therapy and in patients taking Warfarin was considered indicated. We then determined, via chart review, whether each test result led to a change in acute care. Changes in drug treatment that are generally done in the outpatient setting (e.g., treatment of asymptomatic type II diabetes or hypercholesterolemia) were not considered to be a change in acute care that affects patient outcomes. We calculated the proportions of tests that were outside the criteria and the resultant changes in acute care, separately for troponin and the other tests.

## 3. Results

The mean age of the patients was 72 ± 14 years, and 65.2% (307/471) were male. The mean length of hospital stay was 4.3 ± 3.2 days. There were 18,061 laboratory tests performed over 2016 patient days (9.0 tests/day) (Table 2). Tests were frequently ordered in fixed combinations (“panels”). Of the total tests, 79.9% (*n* = 14,427) were outside the criteria, including 70.7% (5702/8069) of the admission tests and 85.8% (8575/9992) of the follow-up tests.

Therefore, altogether, only 20.1% (3634/18,061) of the tests were indicated (1.9 tests per hospital day). The rate of complete blood counts (CBCs) was 0.52, which could be reduced to 0.24 per hospital day by limiting follow-up testing.

There were 1.8% (68/3784) modifications of acute care due to tests ordered in accordance with the criteria. This included blood transfusions (*n* = 9), treatment of diabetes (*n* = 30), treatment of hyperkalemia (*n* = 2), warfarin dose adjustments (*n* = 3), and drug dose reductions in patients with a creatinine value > 1.3 mg/dL (*n* = 14). Included were patients with treatment that could have been given outside the hospital: intravenous iron (*n* = 1), and initiation of treatment for diabetes mellitus (*n* = 9).

Only 0.3% (46/13,806) of tests ordered outside the criteria changed medical care, but did not impact the patient’s acute clinical course. These instances included patients with hemoglobin values between 10 and 11 gm/dL who received intravenous iron (*n* = 2), patients with glucose values < 140 mg/dL who underwent hemoglobin A1c testing and were newly diagnosed with diabetes mellitus (*n* = 19), or were known diabetic patients who had adjustments in their glycemic therapy (*n* = 6), patients with elevated cholesterol values started on a statin (*n* = 7) or who had the dose increased (*n* = 6), a patient with an elevated transaminase who had statins discontinued unnecessarily (*n* = 1) and patients with an initial creatinine level below 1.3 but still led to modifications in diuretic therapy (*n* = 4). Finally, a physician claimed antibiotic therapy was dependent on the C-reactive protein test result (*n* = 1). This was the only case where a physician wrote in the notes that treatment was changed because of a normal test result.

Troponin testing was ordered in 84.9% (*n* = 400) of the patients. In 275 patients (58.8%), the need for a coronary arteriogram was not dependent on the result, including 47.8% (364/761) of the tests ordered (Table 3).

## 4. Discussion

Our study suggests that interventions in our cardiology department could safely reduce troponin testing by nearly 50% and other laboratory tests by approximately 80%. These findings are consistent with the results of a prior before-and-after intervention study conducted in a cardiology department that required physicians to justify laboratory orders [11]. In that study, a reduction of over 70% was observed in the ordering of various laboratory tests, including complete blood counts, troponin, C-reactive protein, liver function tests, and lipids, without any reported serious adverse events or an increase in mortality rates. The current study extends those observations by demonstrating that limiting testing based on predefined criteria did not change acute care management decisions. We suggest that our results support the existing recommendation against routine biochemical testing [12] but we are unaware of international guidelines.

The findings of this study corroborate previous research conducted in departments outside of cardiology, which indicated through chart reviews that testing volumes could be substantially decreased if only tests with the potential to change medical care were ordered. For instance, a study in an academic medical ward in Australia [13] found that 28.6% of tests on admission and 69.3% of tests on follow-up provided no meaningful contribution to clinical management, defined as not altering medical care or being relevant to the patient’s symptoms, or potentially dictating the ordering of new laboratory tests. Another investigation involving 177 patients hospitalized at two Maryland hospitals reported that 49% of admissions and follow-up laboratory or imaging tests did not change medical management [9]. Furthermore, research focusing on patients with community-acquired pneumonia found that limiting testing to only those required to identify all changes in medical care could lead to a reduction in testing by over 80% [10].

We found that troponin tests were done on admission in 84.9% of the patients. We previously reported that the proportion of cardiology patients with troponin tests decreased from 96.7% to 78.2% after recommending testing only those with chest pain or an ischemic electrocardiogram [14]. Nevertheless, tests were still commonly ordered in patients without a suspected myocardial infarction. We also considered that troponin has no clinical utility in patients with chest pain and ST electrocardiographic elevations or new onset congestive heart failure, where a coronary arteriogram is indicated in those with and without elevated troponin test values.

Others justify troponin testing in risk stratification and disease prognostication [15], specifically in patients with congestive heart failure [16,17]. The ordering of troponin and elevated values has been shown previously to predict in-hospital mortality [18,19]. However, even if troponin improves risk stratification, others have pointed out that there is no evidence that this information leads to changes in management and improved outcomes [20,21]. In 2017 Farber et al. [22] emphasized that there are no accepted guidelines for troponin testing in settings other than suspected acute myocardial infarction and no evidence that testing in other disease states alters patient management or outcomes. Recently in 2022, guidelines from the American College of Cardiology and American Heart Association did not recommend ordering a troponin test in patients with congestive heart failure [23]. Recently we reported that in internal medicine patients without chest pain that elevated troponin test results do not provide an independent incremental increase in in-hospital mortality risk, and are associated with repeat testing and unnecessary cardiology consultations without coronary artery interventions [24]. However, it is possible that we did not take into consideration the entire care process, although the charts were carefully reviewed by a cardiologist. Misclassifications, therefore, are likely minimal. Nevertheless, physicians can appropriately order a troponin test outside the suggested criteria if they determine that the result will change the patient’s medical care.

Our study has several limitations. First, it was conducted in a single cardiology department, in a limited number of patients, so extrapolating the results to other departments and healthcare settings should be done with caution. It is also uncertain whether baseline testing rates in our institution are like those in other regions or countries. Nevertheless, our observed testing frequencies are like those reported from hospitals in the United States and Europe. We found an average of 9.0 tests per hospital day (and 0.52 CBC tests per hospital day) in our cardiology ward. By comparison, one analysis of Texas hospitals reported approximately 13 billed laboratory test units per day in patients admitted with bacterial pneumonia [25]. In the Netherlands, internal medicine inpatients averaged about 15 clinical chemistry tests per day [5]. In a medical service of a Maryland academic center, patients underwent a mean of 1.5 CBC tests per day, even after excluding patients with gastrointestinal bleeding, acute renal failure, congestive heart failure, or those transferred to intensive care [26]. Other studies from U.S. hospitals have documented inpatient CBC testing rates ranging from approximately 0.51 to 1.56 per hospital day [27,28,29], which encompasses the rate we observed in our cardiology department.

Secondly, our patient population did not include trauma cases with possible rhabdomyolysis, and we had relatively few patients with severe infections, scenarios in which creatine phosphokinase or C-reactive protein (CRP) testing might be warranted. However, our proposed testing criteria align with expert guidelines that recommend against procalcitonin testing to guide management in patients with community-acquired pneumonia [30]. Current evidence does not support using CRP values to guide the initiation or duration of antibiotic therapy, including in intensive care patients [31].

Thirdly, there were a few instances in which tests outside our criteria did prompt changes in care that have uncertain long-term utility. The most common examples were diagnosing new-onset diabetes mellitus in patients who had an admission glucose level ≤ 140 mg/dL. Some experts consider hospitalization a valuable opportunity to screen for otherwise unrecognized diabetes in such individuals [32]. However, no studies have yet demonstrated that identifying elderly inpatients with diabetes solely through elevated hemoglobin A1c (in the absence of overt hyperglycemia) leads to reductions in mortality or cardiovascular events, or improves overall well-being. It may be more appropriate to address cardiovascular risk factors like mild hyperglycemia in the outpatient setting, where patients can be engaged in long-term care, provide informed consent for screening, and receive appropriate follow-up. This will depend on the availability of testing and treatment in the outpatient setting. Recommendations in the discharge summary for appropriate outpatient testing and treatment might be an effective alternative in some settings.

Fourthly, the results cannot be used to determine if a certain test was warranted in an individual patient where a test might be indicated because of factors not included in this study.

Finally, our results need to be confirmed in other settings and in interventional studies with short-term and long-term follow-up to determine the risks and benefits of implementing the criteria proposed by this study. There has been a call for standardization of intervention methods to reduce inappropriate testing in intensive care units [33], and others have included admission protein electrophoresis, triglycerides, total cholesterol, amylase (if ordered with a lipase test), and liver function tests as appropriate in a university hospital setting [34]. We suggest that the proposed criteria be taken into consideration in future chart reviews and interventional studies to determine the clinical utility and disutility of laboratory testing.

## 5. Conclusions

In conclusion, our study suggests that interventions in cardiology departments could safely reduce troponin testing by nearly 50% and other laboratory tests by approximately 80%. A practical target of 2.0 routine tests per hospital day might be useful for guiding audit and feedback interventions aimed at reducing unnecessary testing.

## Figures and Tables

**Table 1:** Criteria for laboratory testing in the cardiology department.

Test *	Admission	Follow-Up
CRP	No	No
Cholesterol	No	No
CPK	No	No
LDH	No	No
LFTs	No	No
Calcium	No	No
Uric acid	No	No
Blood urea nitrogen	No	No
CBC	Yes	Hemoglobin < 10 gm/dL; Platelets < 100 × 10^9^/L; Neutrophils < 1000 × 10^9^/L or a gastrointestinal bleed
Glucose	Yes	<70 or >140 mg/dL or patients with diabetes mellitus
Sodium	Yes	<135 or >145 mEq/L
Potassium	Yes	<3.5 or >5.0 mEq/L
Creatinine	Yes	>1.3
HbA1c	No	No
PT INR	Before or after treatment with Warfarin	Treated with Warfarin with values outside the therapeutic range
Troponin	Only to rule out an acute myocardial infarction	Only to rule out an acute myocardial infarction

* CRP—C-reactive protein, CPK—creatinine phosphatase kinase.; CBC—complete blood count.; LDH—lactate dehydrogenase, LFTs—liver function tests, HbA1c—hemoglobin A1c.; PT-INR—prothrombin international normalized ratio.

**Table 2:** Laboratory tests not indicated and changes in medical care.

Tests *	Admission NI */Total	Follow-Up NI */Total	Total	Not Indicated	Therapy Changes
CRP	457/457	559/559	1016	1016	1
CBC	0/471	483/569	1040	483	2
CPK	365/365	230/230	595	595	0
Glucose	0/471	435/977	1448	435	2
LDH	446/446	487/487	933	933	0
LFTs	2355/2355	2770/2770	5125	5125	1
Calcium/ Uric acid	732/732	444/444	1176	1176	0
Sodium	0/471	752/921	1392	752	0
Potassium	0/471	778/921	1392	778	0
Creatinine	0/471	469/921	1392	469	4
BUN	471/471	921/921	1392	1392	0
Cholesterol	353/353	173/173	526	526	13
HbA1C	248/248	0/0	248	248	23
PT-INR	275/278	74/99	386	349	0
Total tests *N*/*N* (%)	5702/8069 (70.7)	8575/9992 (85.8)	18,061	14,427 (79.9)	46 (0.3)

* NI—not indicated, CRP—C-reactive protein, CBC—complete blood count, CPK—creatine phosphokinase, LDH—lactate dehydrogenase, LFTs—Liver function coupled tests included serum glutamic pyruvic transaminase, serum glutamic-oxaloacetic transaminase (SGOT), alkaline phosphatase, total bilirubin, and albumin, BUN—blood urea nitrogen, HBA1c—hemoglobin A1C, PT-INR—prothrombin time international normalized ratio.

**Table 3:** Troponin tests.

Diagnosis	Patients *N* (%)	Tested *N* (%)	Total Tests *N* (%)
Indicated troponin tests			
Chest pain, no ischemia	134	129	274
Chest pain, ischemia	62	62	123
Total	196 (41.6)	191 (97.4)	397 (52.2)
The need for a coronary arteriogram not dependent on troponin value			
Chest pain + ST elevation	52	50	102
Congestive heart failure—first episode	3	3	7
Congestive heart failure—recurrent	71	54	98
Elective stent	28	19	34
Arrythmia or heart block	86	58	77
Other presentations	35	25	46
Total	275 (58.4)	209 (76.0)	364 (47.8)
Total	471	400 (84.9)	761

## Data Availability

Data will be available upon reasonable request after approval by the Laniado hospital administration.
